# Fenton’s reaction-based chemical oxidation in suboptimal conditions can lead to mobilization of oil hydrocarbons but also contribute to the total removal of volatile compounds

**DOI:** 10.1007/s11356-019-06547-3

**Published:** 2019-10-26

**Authors:** Harri Talvenmäki, Niina Lallukka, Suvi Survo, Martin Romantschuk

**Affiliations:** Faculty of Biological and Environmental Sciences, Environments and Ecosystems Research Program, University of Helsinki, Niemenkatu 73, 15140 Lahti, Finland

**Keywords:** Fenton’s reaction, Mixed fuel oil contamination, Soil remediation, Rebound concentrations, Chemical oxidation, Contaminant mobilization

## Abstract

Fenton’s reaction-based chemical oxidation is in principle a method that can be utilized for all organic fuel residues thus making it a potential all-purpose, multi-contaminant, in situ application for cases in which storage and distribution of different types of fuels have resulted in contamination of soil or groundwater. Since peroxide breakdown reactions are also expected to lead to a physical transport of the target compound, this secondary physical removal, or rebound concentrations related to it, is prone to be affected by the chemical properties of the target compound. Also, since soil conditions are seldom optimal for Fenton’s reaction, the balance between chemical oxidation and transport may vary. In this study, it was found that, with a high enough hydrogen peroxide concentration (5 M), methyl tert-butyl ether–spiked groundwater could be treated even under suboptimal conditions for chemical mineralization. In these cases, volatilization was not only contributing to the total removal but also leading to rebound effects similar to those associated with air sparging techniques. Likewise for diesel, temporal transport from soil to the aqueous phase was found to lead to false positives that outweighed the actual remediation effect through chemical mineralization.

## Introduction

According to surveys performed in 2013, there are 23,000 sites in Finland that are either suspected to be polluted, declared as such, or have already been treated for contamination. Approximately one in five of these is located in a groundwater area (Pyy et al. 2013). Storage and distribution of fuels is a major contributor to these occurrences even without any major accidents through chronic leaks and small volume spills (Puolanne et al. 1994). In many cases, the contamination is from mixed sources consisting of mid-range to heavier oil fractions and volatile organic compounds (VOCs) with varying characteristics. Gasoline and its additives dissolve in water and therefore pose a risk to groundwater. Remediation of contaminated groundwater is of priority because of high risk of contaminant mobilization. The growing demand for sustainable remediation techniques favours on-site and preferably in situ methods as a way to reduce carbon dioxide emissions from the treatment itself. Under favourable conditions, soil vapour extraction (SVE) of VOCs is an applicable and generally efficient treatment, and in Finland the one most commonly used in situ (Simpanen et al. 2016; OISC 2017). However, since SVE is suitable only for VOCs, contamination from heavier, less volatile hydrocarbon fractions requires alternative treatment methods. These heavy hydrocarbons are also largely immiscible with water, so remediation by solely targeting the aqueous phase is not an option. Less water-soluble diesel fractions are absorbed by soil, and of these the aliphatic compounds are most readily biologically degraded (Kolukirik et al. 2011). As not all fuel residues are in turn expected to react favourably to biological treatment, contamination from mixed sources may lead to longer treatment periods and growing expenses (Simpanen et al. 2018).

One method that in principle allows for simultaneous treatment of multiple contaminants with differing characteristics and in different media is chemical oxidation. It has the potential to be a cost-effective, multi-contaminant method with a reasonable treatment duration. Hydrogen peroxide is the most commonly used oxidant (Innocenti et al. 2014). In a reaction based on Fenton’s chemistry, the chemical breakdown of hydrogen peroxide is catalysed by ferrous iron, and hydroxyl radicals and hydroxide ions are released whilst the ferrous iron is oxidized to ferric iron (Neyens and Baeyens 2003). This first reaction is followed by a series of reactions in which the varying reactive radicals degrade organic compounds, including hydrocarbons (Petri et al. 2011).

When perfect mineralization is achieved, the contaminant is degraded to CO_2_, water and minerals (Petri et al. 2011). The chosen reaction pathway, or rather the balance between different options, is dictated by various parameters and the radical producing pathway is not always favoured. The optimal pH for Fenton’s reaction is near pH 3 (Pignatello et al. 2006), which is lower than that naturally occurring in most soils. Lowering the pH to the desired level in, for example, on-site applications for the aqueous phase can sidestep such difficulties. When dealing with water retained in pore space or heavier oil hydrocarbons absorbed into soil particles, the buffering capacity of most soils tends to become a problem. Near neutral pH, the catalyst is in an insoluble form and the peroxide is consumed by surface iron leading to a negligible amount of radical production in the aqueous phase (Kwan and Voelker 2003). To help widen the pH range in which the catalyst is in a dissolved form, a complexing or a chelating agent such as citrate is introduced (Pignatello et al. 2006). The oxidation state of the catalyst is another factor dictating the efficiency of the reaction, and so the effectiveness of this method is heavily influenced by site-specific soil characteristics (Matta et al. 2007).

The secondary sparging effect of peroxide reactions and their potential to at least temporarily volatize VOCs into the pore space has also been noted. Volatilization connected to chemical oxidation is generally recognized, but more as a potential risk during treatment rather than an added benefit (Petri et al. 2011), even if the two are combined. Gas release during the hydrogen peroxide breakdown reaction can be intense and thus have an effect on volatile compounds similar to that of air sparging methods.

If this physical effect is of similar scale and magnitude as reductions through chemical mineralization, this would have two consequences: what could be called “peroxide sparging” could be used as a remediation method for sites with VOC-contaminated groundwater even when conditions for chemical oxidation appear unfavourable. Since one of the challenges of air sparging lies in that its radius of influence is limited due gas escaping the soil, the ability to generate a similar but delayed sparging effect through distribution of fluids instead, should prove beneficial. If not applicable as a stand-alone method per se, secondary volatilization could still add to the total efficiency of Fenton’s oxidation. The total effect could in this case be improved and the environmental risks lowered by simultaneous collection of the VOC emissions using soil vapour extraction.

Another consequence is that this process could lead to rebound phenomena not associated with chemical oxidation: with non-volatile compounds such as light fuel oils, sparging would assumedly only lead to a temporary mobilization and possible displacement of the contaminant within the site. Also for VOCs, whilst volatilization is hoped to result in complete physical removal of the compound, a rebound effect may also be expected once the gas dissolves back into the aqueous phase. In which manner, and under what circumstances these effects influence the course of Fenton has not been thoroughly tested at different scales (Petri et al. 2011).

Methyl tetra-butyl ether (MTBE) meets all of the requirements for a contaminant that is treatable with air sparging (Spencer et al. 1988). In Finland, MTBE has been used as a fuel additive since 1991 (Tidenberg et al. 2009) and its use has not decreased due to regulation in a manner similar to the USA (Lindsey et al. 2017). The MTBE concentration used in gasoline sold in Finland is higher than in the EU generally, and is similar to the 10% level used in the USA (Tidenberg et al. 2009). In addition to the concerns of groundwater contamination by MTBE in Finland, it is an ideal compound for tracing rebounds from the gaseous phase. Since it has a relatively high solubility, the amount appearing as non-aqueous phase liquid (NAPL) is kept low. The probability of rebound concentrations is generally acknowledged to be more connected to the presence of NAPLs and changes in the groundwater level brought upon by the remediation techniques themselves (Bass et al. 2000).

The roles played by H_2_O_2_, Fe (III) and Fe(II) catalysts, and citrate chelate in Fenton’s chemistry–based remediation were tested in a laboratory scale experiment on MTBE-spiked water retained in the pore space of sand/gravel. This was done to study the extent to which the concentrations of each additive would dictate the removal of MTBE from the aqueous phase as assessed from general VOC levels measured with a photoionization detector (PID). In another test focusing on the composition of the gaseous phase, it was studied whether in some circumstances successful removal of MTBE could be attributed to volatilization rather than to chemical oxidation.

As a transitional step between the laboratory tests and a site treatment, the method was tested on MTBE-spiked pore water at a lysimeter station in 1–2 m^3^ scale. At this scale, the technical issues of reagent additions could also be addressed. In the lysimeter scale experiment, the treatment was also tested on soil with aged diesel contamination with interest in the differences in performance level and mechanism. Aged diesel was therefore selected as another focus compound to study the efficiency of in situ chemical oxidation as a multi-fuel remediation method and to see in which way a possible sparging effect complicates these situations with contamination from a combination of gasoline and diesel components. In Finland from 2007 onwards, the focus of in situ remediation has spread to diesel-range oils (Nikunen et al. 2017) partly allowed by their low mobility and hence the low risk level of the contaminant. If the mobility is positively affected by the aforementioned mechanisms, these risks are heightened.

Questions we set to answer were as follows:

1. What are the influences of hydrogen peroxide concentration, catalyst concentration, the oxidation state of the catalyst and citrate chelate addition on the performance efficiency of treatment with high concentration of H_2_O_2_ on MTBE-contaminated pore water in different scales?
2. What are the conditions in which secondary physical mechanics of these reactions could be seen as contributing to the total reduction of the contaminant?
3. What is the efficiency of the peroxide treatment on MTBE-contaminated pore water and aged diesel–contaminated soil in lysimeter scale experiments, and what is the role of the rebound mechanism associated with the sparging effect in both cases?

## Materials and methods

### Test soil

The soil used in all tests was 60% sand (grain size 0.06–2 mm), 40% gravel (< 8 mm) with organic matter content of 8.6 g kg^−1^ dw as measured according to standard method SFS 3008. The amount of elemental Fe in the soil was 14.2 g kg^−1^ dw as measured with MARS (MARS-6, CEM Corporation, 200 °C temperature, hold time 20 min). Soil pH was approximately 5.9 as measured with standard method ISO 10390. The moisture level varied at different instances due to outdoors storage, but was measured at every relevant stage by collecting and drying samples overnight in 105 °C temperature.

The minimum oxidant demand for soil was estimated with permanganate as described by Haselow et al. (2003). An 800 mg dose of KMnO_4_ was mixed with 100 g of soil and 16 g of ultra-pure (mQ) water for similar moisture content used elsewhere in the experiment, and left to react for 48 h in 20 °C. The remaining amount of permanganate was measured by titration with a 0.01 M Na_2_S_2_O_3_ solution. Equal mass of KMnO_4_ was titrated separately for standardization. The soil with and without the maximum dose of catalyst used in the experiments (20 mM Fe(III)sulfate) were tested, both as three replicas, and the results were reduced from that of a blank sample, with only the permanganate and water added. The minimum soil oxidant demand of 100 g of soil without contamination or additives was 86 ± 2 mg (average ± 95% confidence interval) of permanganate, reduced to 51 ± 4 mg with the dose of Fe(III)sulfate.

### Reagents

H_2_O_2_ at 35% and 50% solutions were purchased from Bang & Bonsomer Group oy in Finland. Fe(III)sulfate (Fe_2_(SO_4_)_3_ × H_2_O) and sodium citrate dihydrate ≥ 99%, FG were purchased from Sigma-Aldrich Germany and Fe(II)sulfate (FeSO_4_*7H_2_O) from Honeywell Fluka Germany. MTBE, purity level ≥ 99%, was purchased from Merck Schuchardt OHG, Germany.

### Laboratory scale survey on the impact of different additives on MTBE-spiked pore water

Crude numerical data on the effect of different additive concentrations, namely hydrogen peroxide, Fe catalysts with varying oxidation state, and citrate as the chelate, on hydrocarbon removal efficiency was gathered from a small-scale test utilizing a handheld PID-metre (Microtip Photovac Mp-100 photoionization measuring general non-specific VOC levels). This allowed for a large number of parameters to be tested, as well as several replicas of each individual combination at a relatively low expense.

This test followed the assumption that, at the minor scale, the level of VOCs measured with the PID would also reflect the concentrations of MTBE in the aqueous phase, which would enable a rough comparison between different treatments needed for planning the larger scale applications.

The effect of hydrogen peroxide and catalytic Fe(III) concentrations, and the addition of trisodium citrate chelate on VOC levels were tested with pore water spiked with MTBE (750 mg L^−1^). All selected H_2_O_2_ and Fe(III)sulfate concentrations were tested both with and without the citrate addition (Table 1). The choice of Fe(II)sulfate as the alternative catalyst was tested in 20 mM concentration.

**Table 1:** The tested additive concentrations in the lab scale preliminary pilot. All listed combinations were tested as three replicas

H_2_O_2_ (M)	5	5	5	5	5	2	2	2	2	1	1	1	1	0.5	0.5	0.5	0.5
Fe(II)SO_4_ (mM)	20	−	−	−	−	−	−	−	−	−	−	−	−	−	−	−	−
Fe(III)_2_(SO_4_)_3_(mM)	−	20	2	0.6	−	20	2	0.6	−	20	2	0.6	−	20	2	0.6	−
Citrate (50 mM)	+/−	+/−	+/−	+/−	+/−	+/−	+/−	+/−	+/−	+/−	+/−	+/−	+/−	+/−	+/−	+/−	+/−

MTBE-spiked ultra-pure water (mQ) together with 1000 ml of soil was poured into 1-L glass bottles. The PID values and soil temperature were measured first at 15 min intervals and with lengthening gaps from 60 min onwards. The last measurements were performed on the next working day, which would in some cases mean 48+ h or 72+ h after the injections, rather than the usual 24+ h (48+ h, 5 M H_2_O_2_ + 0.6 mM Fe(III)sulfate + citr.; 2 M H_2_O_2_ + 0.6 mM Fe(III)sulfate + citr.; 72+ h, 1 M H_2_O_2_ + 2 mM Fe(III)sulfate; 2 M H_2_O_2_ + citr.; 1 M H_2_O_2_ + 2 mM Fe(III)sulfate + citr.; 5 M H_2_O_2_ + 2 mM Fe(III)sulfate + citr.; 2 M H_2_O_2_ + 20 mM Fe(III)sulfate + citr.). This choice for timeline was based on preliminary test in tenfold scale, in which peroxide was consumed within the first 24 h to low enough concentrations where neither chemical oxidation nor enhanced volatilization would occur.

Since iron additives and citrate both affected the pH, this parameter was tested with every possible combination of additions. From this analysis, the effect of H_2_O_2_ on soil pH was excluded since the effects of this addition as such on pH would not be differentiated from those caused by the breakdown reaction. Only the change in soil pH caused by peroxide at the highest concentration (5 M) was measured at 1 h and 3 h after injection.

### Analysis of the volatile compounds and breakdown products in the aqueous phase

The validity of the PID-method was tested in an experiment utilizing an absorbent (Anasorb 747, SKC 226-81A) coupled with a Markes Acti-Voc pump. The aim was to calibrate the general VOC results generated by the PID with the results of individual compounds during the first hours of the reaction and hence reveal whether the same removal mechanism could be identified at different pH values measured in the PID test. The results were used as a crude estimate of mass balance between the aqueous and gaseous phases. In treatments with expansive gas formation, vapours were exiting the system through joints.

For the experiment, 20-L PVC buckets with lids were used. The experiment volume was scaled up tenfold from the PID test, and the spiked MTBE concentration was decreased from the PID test, to 15 mg L^−1^. The reduction in concentration was done to play down the volatizing effect of the spiking event itself, and the scale was increased to ensure detectable concentrations from the headspace even within brief intervals.

The treatments with 5 M H_2_O_2_, both with and without 20 mM Fe(III)sulfate addition, were compared with a water control in regard to the composition of VOCs at three different time points, 15 min, 60 min and 240 min. At each time point, a 1.5-L sample was collected over 15 min from the 12.3 L headspace of the bucket. Each sample was taken from a separate bucket to limit leakage of vapours until the sampling event. One additional sample was withdrawn after 21 h from the treatment without the catalyst, to compare particular values measured in the PID test. After 20+ h, a water sample was withdrawn to determine if decreasing ambient VOC levels were connected to decreasing concentrations in the aqueous phase. This water sampling was postponed due to the gas producing reactions in the aqueous phase during the time of the final headspace sampling. Putative chemical breakdown products of MTBE were monitored both in the air and in the water.

### Lysimeter scale pilot tests

Pilot-scale tests for aged diesel contaminated soil and MTBE contaminated pore water were performed in 1.6 m^3^ metal lysimeters at the SOILIA field research station in Lahti in May–June in years 2016 and 2017 respectively. The lysimeters have been described in more detail earlier (Simpanen et al. 2016). The purpose of these studies was to detect scale-dependent phenomena moving from laboratory to field scale, concerning both the injection protocol and the level of success in treating MTBE as a VOC entirely dissolved in the aqueous phase, and aged diesel that was absorbed into soil particles and having lower relative water solubility.

### MTBE pilot test

MTBE-spiked porewater (750 mg L^−1^) in 1 m^3^ of soil was treated in a three-phase experiment (Table 2). The water content of the soil was measured with a moisture sensor as an average between several spots whilst building the soil column for the lysimeter. Upon injection into the soil, the additives were diluted to half of the original concentration. In the treatment test, MTBE in diluted H_2_O_2_, and in the control test, MTBE in a corresponding volume of water, were poured into the soil columns. For desired peroxide concentrations, total injected volume was increased by 40 L after each step due to heightened dilution in the increased pore water volume, and this increase in volume was compensated for by addition of new MTBE. The upper portion of the soil column remained non-saturated for the whole treatment period.

**Table 2:** The experiment protocol for MTBE-spiked pore water in sandy soil. Different treatments nd concentrations of additives in the two lysimeters during the three stages

lysim. 1	lysim. 2
1 m^3^ (1.4 t) soil	1 m^3^ (1.4 t) soil
Treatment 1	Treatment 1
MTBE (240 g, 750 mg L^−1^)	MTBE (240 g)
	H_2_O_2_ (1 M in total volume)
Water (160 L added; 320 L total volume)	Aqueous phase (160 L; 320 L)
Treatment 2	Treatment 2
MTBE (30 g, 750 mg L^−1^)	MTBE (30 g)
	H_2_O_2_ (2 M in total volume)
Water (40 L; 360 L)	Aqueous phase (40 L; 360 L)
Treatment 3	Treatment 3
MTBE (30 g, 750 mg L^−1^)	MTBE (30 g)
H_2_O_2_ (2 M in total volume)	H_2_O_2_ (2 M in total volume)
Fe(II)SO_4_ (20 mM in total volume)	
Aqueous phase (40 L; 400 L)	Aqueous phase (40 L; 400 L)

During the third phase of the experiment, in the second lysimeter the control treatment was replaced with a test to examine Fe(II)–enhanced peroxide treatment. This was done to study the effect of catalyst addition on reaction intensity and its radius of influence. Fe(II)sulfate was weighed into three vertical holes (depth 50 cm) in the soil column prior to peroxide injection.

Water samples were withdrawn from the bottom valves of the lysimeters, which remained otherwise closed. Soil temperature readings and ambient VOC concentrations, measured with a handheld PID, were taken from vertical holes drilled into the soil column. At the end of the three-phase experiment, after the water phase had been drained, the soil column was sampled in four vertical sub-samples for VOC analysis.

### Diesel lysimeter test

Chemical oxidation of diesel-contaminated soil and the role of additional catalyst and chelate were tested in a separate pilot test with soil with aged diesel contamination. The experiment protocol is included in Table 3.

**Table 3:** The experiment protocol for aged diesel–contaminated soil. Different treatments and concentrations of additives during the two stages (X = added, o = not added) (^a^ final concentration in total water volume; ^b^ none added, diluted to final concentration)

Soil				
1.5 m^3^ (2 t)				
	H_2_O_2_ + citr. + Fe(II)	H_2_O_2_ + citr.	H_2_O_2_	Control
Treatment 1				
H_2_O_2_ (3.1 M^a^)	X	X	X	o
trisodium citrate (61 mM^a^)	X	X	o	o
Fe(II)SO_4_ (24mM^a^ )	X	o	o	o
Aqueous phase (added 330 L; total 390 L)	X	X	X	(420 L; 470 L)
Treatment 2				
H_2_O_2_ (2.5 M^a^)	X	X	X	o
trisodium citrate (47 mM^b^)	X	X	o	o
Fe(II)SO_4_ (19 mM^b^)	X	o	o	o
Aqueous phase (added 110 L; total 500 L)	X	X	X	o

Approximately 2 tons (dw) of aged diesel–contaminated soil (1000 mg kg^−1^ dw) with 50 L of water retained, was weighed into the lysimeters and sampled for the original concentrations of oil hydrocarbons. The samples were withdrawn with an auger from the top from two depths, 0–50 cm and 50–100 cm, in three separate drillings.

Solid compounds Fe(II)sulfate and citrate were first added to the soil column dissolved into 50 L of water. H_2_O_2_ additions were then performed twice with a 3-week interval, once the peroxide concentrations were under the detection limits as measured with peroxide strips (Quantofix® Peroxide).

During the treatment the water surface was above the soil surface and soil samples were taken only from the surface soil (5–10 cm) in 3–6 replicates. Water samples were withdrawn either from the surface or from the bottom valves depending on whether the peroxide reactions were pushing the plume up or allowing it to descend. Soil samples were prepared for oil analysis within 4 h. Soil temperature was measured during each site visit. After the experiment was concluded, the soil column was sampled from depths 0–5 cm, 5–50 cm, 50–100 cm and 100–150 cm, with three individual samples from each depth.

### Analyses

The VOC levels in water from the laboratory and pilot-scale studies were analysed at the accredited Eurofins Environment Testing Finland Oy laboratory in Lahti, using method RA4050 (based on modified ISO 11423-1 and mod. EN ISO 10301) utilizing HS/GC/MS, and diesel concentrations in water according to standard method SPFS-EN ISO 9377-2. Diesel in soil was analysed according to ISO 16703:2004 by the research group.

The absorbent collector was analysed at the Finnish Institute of Occupational Health, with the accredited method KEMIA-TY-006 utilizing GC-MS (method based on standard method SFS-3861, and when applicable, on methods NIOSH 1003, 1300, 1400 and 1500–1501 and the 3M Technical Data Bulletin 1028 sheet). Soil pH was measured according to method ISO 10390.

### Statistical analysis

Statistical analysis was performed on the data received from the laboratory scale survey on the impact of different additives on MTBE-spiked pore water. As the requirement of homogeneity of variances of a parametric ANOVA could not be met even with data transformations, the effects of additives and their combinations on general VOC levels, as measured with PID, were analysed using a three-way Kruskal-Wallis test.

A pairwise comparison between effects of Fe(III) and Fe(II) was done by comparing the differences in ordinal number sums for different treatments to a computed yardstick value W_I_ for risk level *α* = 0.05 (formula 1; Ranta et al. 2012). Differences larger than *W*_*I*_ were considered significant.

1 $$ {W}_I={Q}_{\alpha \left(I,\infty \right)}\sqrt{\frac{n_0\left({n}_0I\right)\left({n}_0I+1\right)}{12}} $$

where

*Q*_*α*_: value from Studentized range *q* table for the chosen risk level
*n*_*0*_: number of observations from a particular group
*I*: number of groups compared

In the lysimeter tests, 85% confidence intervals were used to approximate risk level *α* = 0.05 (Cumming 2009; Paaso et al. 2017; Mikola et al. 2018).

## Results

### Laboratory scale survey of impact of different additives on MTBE-spiked pore water

**Fig. 1:**
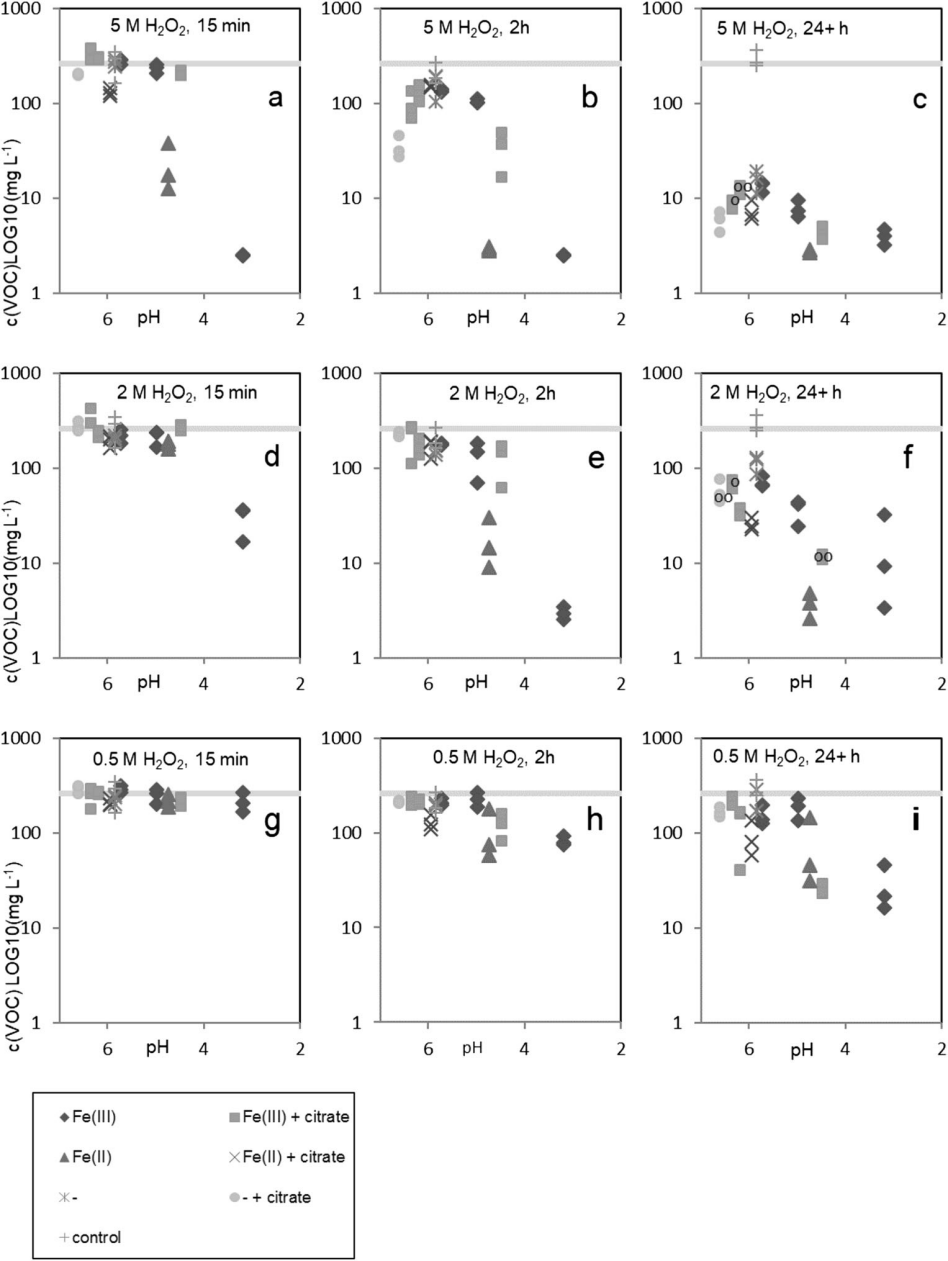
**a**–**i** VOC concentrations as measured with PID in relation to soil pH after Fe and citrate additions. With H_2_O_2_ concentration 0.5 M, 2 M and 5 M at sampling instances 15 min, 2 h and 24+ h. LOG10 transformed y-axis. Grey line indicates original level (average between all treatments, 85 %, confidence interval indicated by the line width) and does not correspond with soil pH. Several treatments appear at different positions on the x-axis because several different concentrations were tested. The group of results marked ‘o’ relate to 48+ h values and the ones marked ‘oo’ to 72+ h values

In the small-scale test with MTBE-spiked pore water, the effect of H_2_O_2_, catalyst and citrate additions on general ambient VOC levels as measured with PID, were followed for at least 24 h. Both the catalyst and citrate affected the initial soil pH, but in opposite fashion: 20 mM dose of Fe(III)sulfate lowered the soil pH from 5.9 to 3.2 and with only the alkalizing effect of citrate, the initial soil pH was increased to 6.6. With all possible combinations of additives, the soil pH was hence between the aforementioned extremes. The largest peroxide dose alone decreased pH to level 5.6. These changes were likely contributing to the performance level in each individual case as the results were obtained under highly varied pH conditions (Fig. 1).

Within the studied range of additive concentrations, increasing H_2_O_2_ and catalyst doses affected the removal rate positively; whilst citrate addition was found to have the opposite effect (Table 4). In each case of a positive effect, a push towards more optimal pH conditions had been achieved with the additions and vice versa. In the presence of peroxide, the effect of Fe(III)sulfate concentration was statistically significant after 15 min, but the effect of increasing peroxide concentration alone was significant only after 120 min onwards. According to this, excessive dosage of the catalyst was the best indicator of the initial reaction intensity.

**Table 4:** The effect of additive concentrations on ambient VOC levels. The degrees of freedom (df) and H statistics of three-way Kruskal-Wallis tests of the effects of H_2_O_2_ (doses of 0.5, 1, 2 and 5 M), Fe(III)sulfate (0, 0.6, 2 and 20 mM) and citrate (0 and 50 mM) on general VOC levels at different time points as measured with PID (*P < 0.05, **P < 0.01, ***P < 0.001)

		Time (min)								
		0	15	30	45	60	90	120	180	1–3 d
Treatment	*df*	*H*	*H*	*H*	*H*	*H*	*H*	*H*	*H*	*H*
H_2_O_2_	4	5.4	3.4	2.9	1.7	1.7	9.2	24.2***	43.6***	59.7***
Fe(III)	4	9.3	46.7***	48.6***	41.3***	45.7***	53.2***	43.7***	41.1***	34.5***
Citrate	1	4.8*	12.3***	17.3***	19.7***	16.2***	14.9***	7.6**	5.9*	0.1
H_2_O_2_ × Fe(III)	12	20.8	9.8	7.2	9.2	11.1	7.0	6.9	6.6	7.3
H_2_O_2_ × citrate	3	0.8	5.9	3.6	4.0	7.2	7.1	4.6	6.0	1.0
Fe(III) × citrate	4	14.2**	2.7	3.6	5.8	5.9	3.0	6.2	3.3	4.1
H_2_O_2_ × Fe(III) × citrate	12	22.6*	13.4	9.7	14.3	12.2	7.9	7.3	4.3	2.5

The differences in conditions affected the outcome in a more apparent fashion in the early hours of the experiment, and with low H_2_O_2_ concentrations. With peroxide concentration 0.5 M these short-term differences could be used as predictions of the total effect (Fig. 1h and i). With 5 M concentration, however, the effect of all parameters, that is, concentrations of other additives and pH, was cancelled out to a high degree and the final removal rate was in each case in the 90% range.

**Fig. 2:**
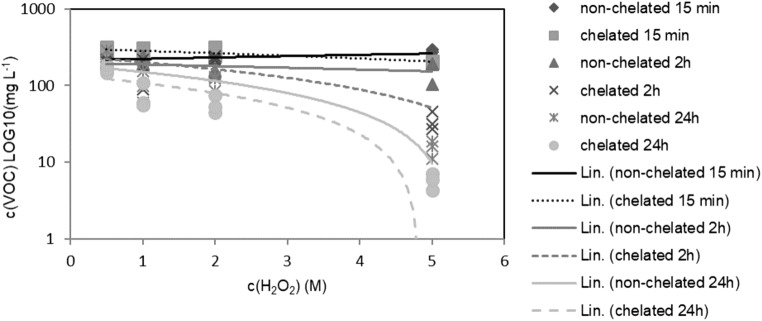
The correlation between peroxide concentration and VOC (PID) concentration with and without chelate when additional catalyst was not added. Results from measurements 15 min, 240 min and 24+ h after peroxide injections. Lin refers to linear regression. LOG10 transformed Y-axis

The negative effect of citrate addition on the reduction rate of VOCs was apparent within the timeframe of 0–180 min. However, when the effect of catalyst addition was removed, citrate appeared to increase the correlation between peroxide concentration and the VOC removal rate, suggesting that without chelate, excessive doses of peroxide were not beneficial in regards to the rate of contaminant removal (Fig. 2).

Fe(III)sulfate and Fe(II)sulfate were tested in quantities of mole of product rather than mole of Fe or SO_4_. The effect of the highest dose (20 mM) of Fe(II)sulfate was found to not be statistically different from those of the highest two Fe(III)sulfate doses (2 and 20 mM). With a 5 M H_2_O_2_ concentration, reductions with the highest doses of catalysts were of similar magnitude regardless of their oxidation state (Fig. 1c). In addition to the differences in the amount of ions introduced in each case, Fe(II)sulfate also had weaker effect on the initial soil pH, meaning that with Fe(II) the positive effect was to a lesser extent connected to its role as an acidifying agent.

### Laboratory scale test on VOC composition on Fenton-treated MTBE-spiked pore water

In the test the composition of VOCs and indicators of chemical oxidation of MTBE in the aqueous phase were compared in three different cases, with 5 M H_2_O_2_ dose both with and without additional 20 mM Fe(III)sulfate catalyst, and with non-treated soil. Of the spiked amount, approximately 50% was detected in the aqueous phase of the control treatment, with 15% of the reduction caused by dilution.

The spiked amount of 15 mg L^−1^ of MTBE was reduced to below the level of quantification (0.5 μg L^−1^) with 5 M H_2_O_2_ and 20 mM Fe(III)sulfate (Table 5). Results were hence below 0.007% from that in the control study. Chemical oxidation as the removal mechanism was implied by the presence of breakdown products such as tert-butyl formate (TBF) in the gaseous headspace and acetone in the aqueous phase (Burbano et al. 2008). The concentration of TBF peaked already within the 4 h monitoring period.

**Table 5:** Concentrations of MTBE and its breakdown products in air and in the aqueous phase. The percentage corresponds with the MTBE concentration in headspace related to the mass initially added

		Ambient headspace				Aqueous phase		
	Time	C9–C11 cyclic	MTBE	TBF	Acetone	MTBE	Acetone	Methyl-ethyl-ketone
		(mg m^−3^)	(mg m^−3^)	(mg m^−3^)	(mg m^−3^)	(μg L^−1^)	(mg L^−1^)	(mg L^−1^)
5 M H_2_O_2_+20 mM Fe(III)_2_SO_4_	15 min	< 0.3	120 (5 %)	10	< 1			
	1 h	< 0.3	94 (4 %)	14	< 1			
	4 h	< 0.3	1.8 (0.1 %)	1.9	< 1			
	20 h					< 0.5		< 0.05
5 M H_2_O_2_	15 min	< 0.3	430 (18 %)	< 0.6	< 1			
	1 h	< 0.3	320 (13 %)	< 0.6	< 1			
	4 h	< 0.3	160 (7 %)	1.6	< 1			
	20 h	< 0.3	7.6 (0.3 %)	2.8	1.9	8.8		0.10
Control	15 min	0	160 (7 %)	< 0.6	< 1			
	1 h	0	240 (10 %)	< 0.6	< 1			
	4 h	2.9	290 (12 %)	< 0.6	< 1			
	20 h					7700		< 0.05

Without added catalyst, the end concentration in the aqueous phase was 0.1 % of the level in the control study. Concentration of MTBE in the headspace surpassed the control treatment level during the first hour, without any breakdown products present in concentrations above the levels of quantification. This would suggest that enhanced volatilization was adding to the removal efficiency, at least during the initial stages. Additionally, the presence of TBF and acetone in the gaseous phase and acetone in the aqueous phase demonstrated that chemical oxidation was still occurring without added catalyst at pH 5.6 (Table 5).

Volatilization could not be ruled out with the addition of catalyst, but its effect was at least cancelled out by reduction of the contaminant in the aqueous phase through chemical mineralization. Its role in total reduction was therefore negligible in comparison with the soil mineral–catalysed reaction. The balance between the two removal mechanisms was therefore found to be affected by adding catalyst, soil pH or both together (Table 5).

### MTBE lysimeter pilot

**Fig. 3:**
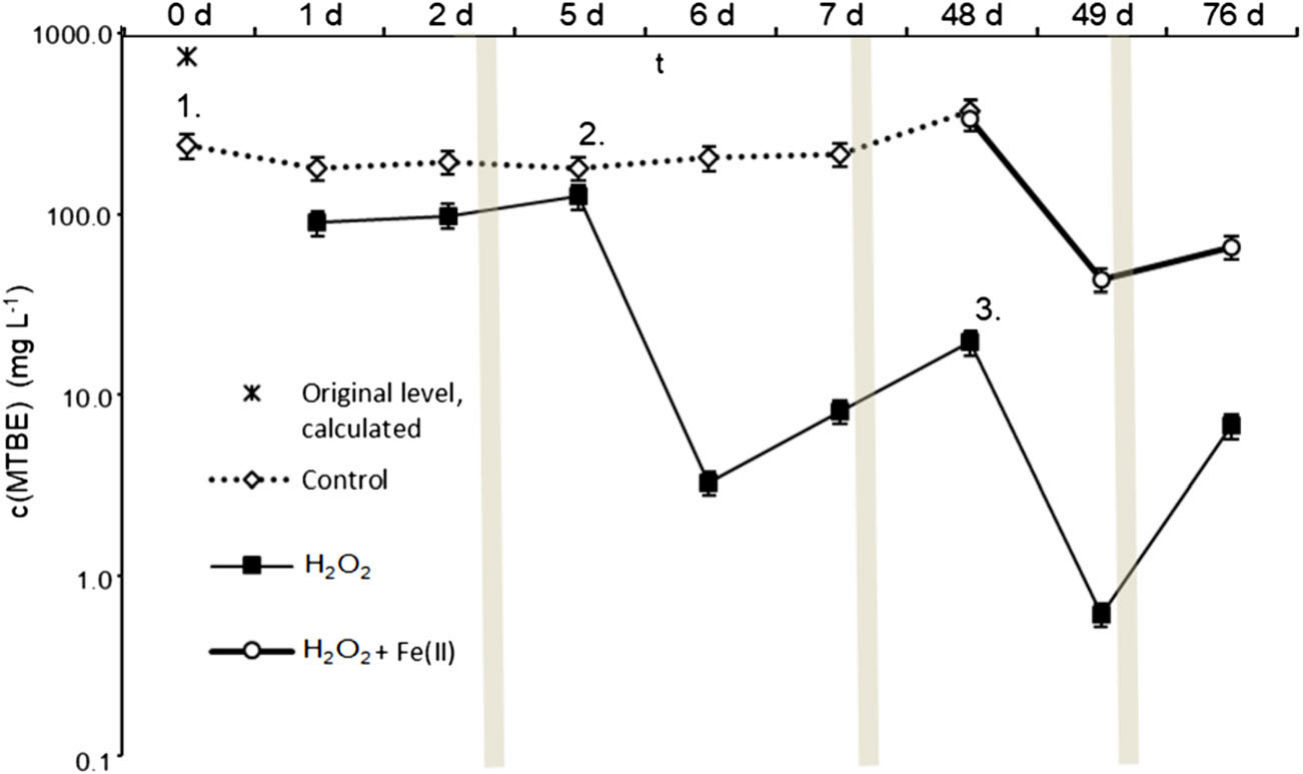
The effect of treatment on MTBE concentrations in pore water in relation to time after the initial addition. Results show average ± 85 % confidence intervals. Grey vertical lines indicate non-uniform timeline. Log10 transformed y-axis. The treatment numbers correspond to those in Table 2

**Fig. 4:**
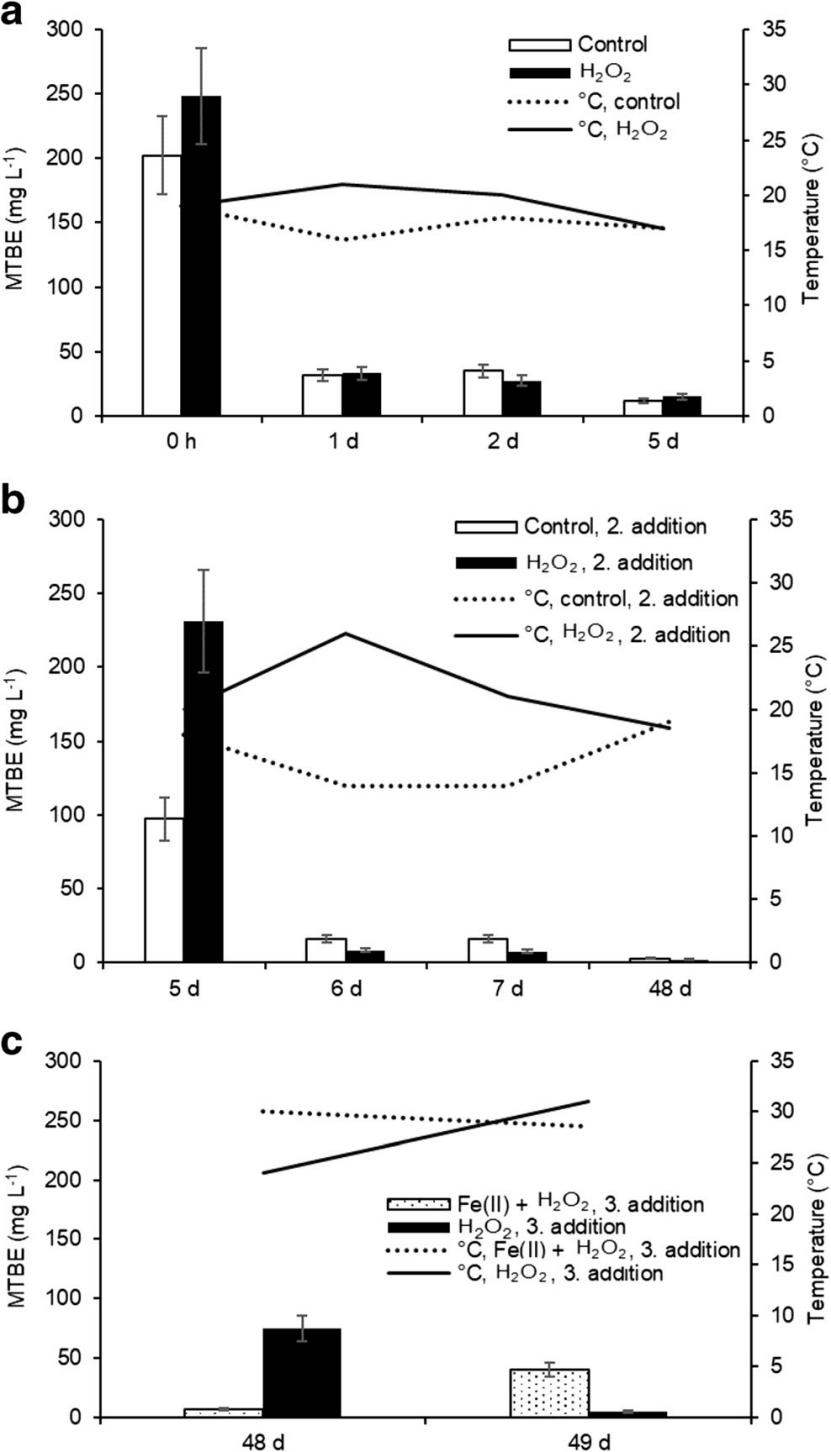
a–c The effect of treatment on ambient VOC concentrations and soil temperature. Bar graphs show the ambient general VOC levels (mg L^−1^) (average ± 85 % confidence interval, *n* = 7) and line graphs the soil temperature (°C) at different measuring instances after the first (a), second (b), and third (c) additions. Prior to the injections, the background level was in each case 2–4 mg L^−1^

In the lysimeter scale experiment, the peak MTBE concentration measured from the pore water in the control treatment during the span of the treatment was only approximately 50% of the added amount (Fig. 3). This concentration peak in the control treatment was documented only after the waiting period (43–48 days from the second and first injection respectively) since during the first days after each injection surface soil bound water was still likely acting as a source for MTBE. Since high PID values were recorded also with non-treated soil, the injection event itself was contributing to the reduction through volatilization (Fig. 4a–c).

A single addition of H_2_O_2_ to reach 1 M concentration led to an initial 50% reduction in MTBE concentration when compared with concentrations in the control treatment during days 1 and 2 (Fig. 3). When the process was monitored 3 days later, on day 5, a rebound effect was observed and the apparent total removal had decreased to 31%.

A second injection of H_2_O_2_, leading to a doubled end concentration (2 M) in pore water resulted in a further 97% reduction in MTBE–concentrations, totalling now a 98% reduction from the concentrations in the control treatment. After a waiting period of 41 days, the end concentrations were still 95% lower than the level in the control study. One additional injection with a similar 2 M end concentration led to a further 66% reduction, now 99.3% in total from the last measured value in the control treatment. From the vertical soil samples taken at the closure of the experiment, MTBE concentrations were found to correlate with soil moisture level (*R* = 0.99).

The portion of the rebound of the total reduction was found to increase with lowering MTBE concentration. The rebound from day 1 to day 5 was approximately 40% of the magnitude of the initial reduction whereas the two following treatments resulted in 600% (day 6 day 48) and 1100% rebounds (day 49 day 76) respectively.

The use of additional Fe(II) was found beneficial considering that the resulting 88% drop in MTBE concentrations within the first 24 h was the steepest recorded decline during the experiment. However, when working with the additional catalyst, the temperature at the surface rose to 100 °C and some portion of the treatment solution hence evaporated before reaching the target depth. This could be verified with peroxide strips from water samples taken from the bottom valves, as the peroxide concentrations were below the level of quantification (1 mg/l).

In the lysimeter scale, the concentrations in the aqueous phase were no longer found to affect the ambient VOC levels. In the control treatment VOC levels fell to below 20% of the initial level even within 24 h from the injection (day 2, day 6) even if similar reductions in concentration was not detected from the water samples. The spiking event itself was observed to increase the VOC levels, but the peroxide injection still resulted in elevated values in all cases in comparison with the control treatment whereas these differences were still of the same order of magnitude, 100–300 mg L^−1^ (Fig. 4a–c).

### Diesel lysimeter test

**Fig. 5:**
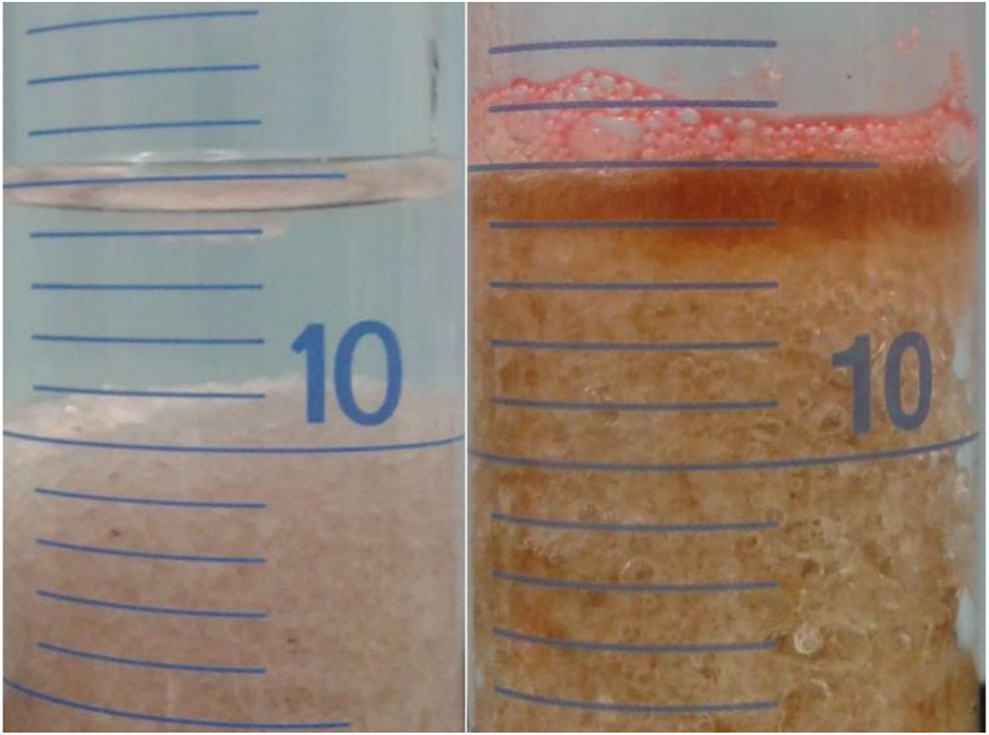
The effect of peroxide on quartz sand spiked with red dyed diesel, before (left) and after (right) peroxide addition. Due to gas production the soil bound diesel is concentrated on the soil surface

When chemical treatment was performed on aged-contaminated soil, addition of peroxide was found to raise the water layer through gas formation and lead to temporal mobilization of diesel from the top soil to the water phase. The vertical range of the mechanism was not determined but in small scale both the effect of the gas formation on the plume and the mobilization could be demonstrated (Fig. 5). The effect was seen in the way the oil hydrocarbon concentrations in the top soil dropped considerably after the injection, but in some cases slowly returned to the initial state once the reaction began to wane (Table 6). Addition of chelates affected the reaction intensity positively and also led to faster reaction burnout as measured from water temperature, whereas when chelation was combined with Fe(II) these attributes were not further affected.

**Table 6:** Oil hydrocarbon concentration in the surface soil and water samples. Water samples could in most cases be obtained either from surface or from the bottom valves depending on whether the formation of gases was lifting the plume. Concentration in mg kg^−^1 refers to kg of dry weight.

	H_2_O_2_				H_2_O_2_ + citrate				H_2_O_2_ + citrate+Fe(II)			
Treatment/days after treatment	Surface soil	Surface water	Bottom water	*t*	Surface soil	Surface water	Bottom water	*t*	Surface soil	Surface water	Bottom water	*t*
	mg kg^−1^	mg L^−1^	mg L^−1^	°C	mg kg^−1^	mg L^−1^	mg L^−1^	°C	mg kg^−1^	mg L^−1^	mg L^−1^	°C
1/0	990 ± 70	…	…	15	960 ± 120	…	…	15	940 ± 40	…	…	17
1/1	590 ± 10	76	…	35	480 ± 80	120	…	45	350 ± 40	210	…	46
1/2				34				27				28
1/4	450 ± 10	210	…	18	620 ± 50	64	…	15	480 ± 60	440	…	15
1/8				15				12				11
1/10				14				13				11
1/14	440 ± 80	…	0.49	14	900 ± 50	…	<0.05	13	670 ± 80	…	1.5	13
2/1	530 ± 60	120	…	21	410 ± 40	100	…	43	210 ± 30	320	…	45
2/5				18				15				15
2/7	790 ± 80	…	1.0	…	570 ± 20	…	0.21	…	450 ± 40	170	1.2	…
2/14	710 ± 50	…	0.76	…	970 ± 80	…	0.10	…	360 ± 10	…	0.46	…

**Fig. 6:**
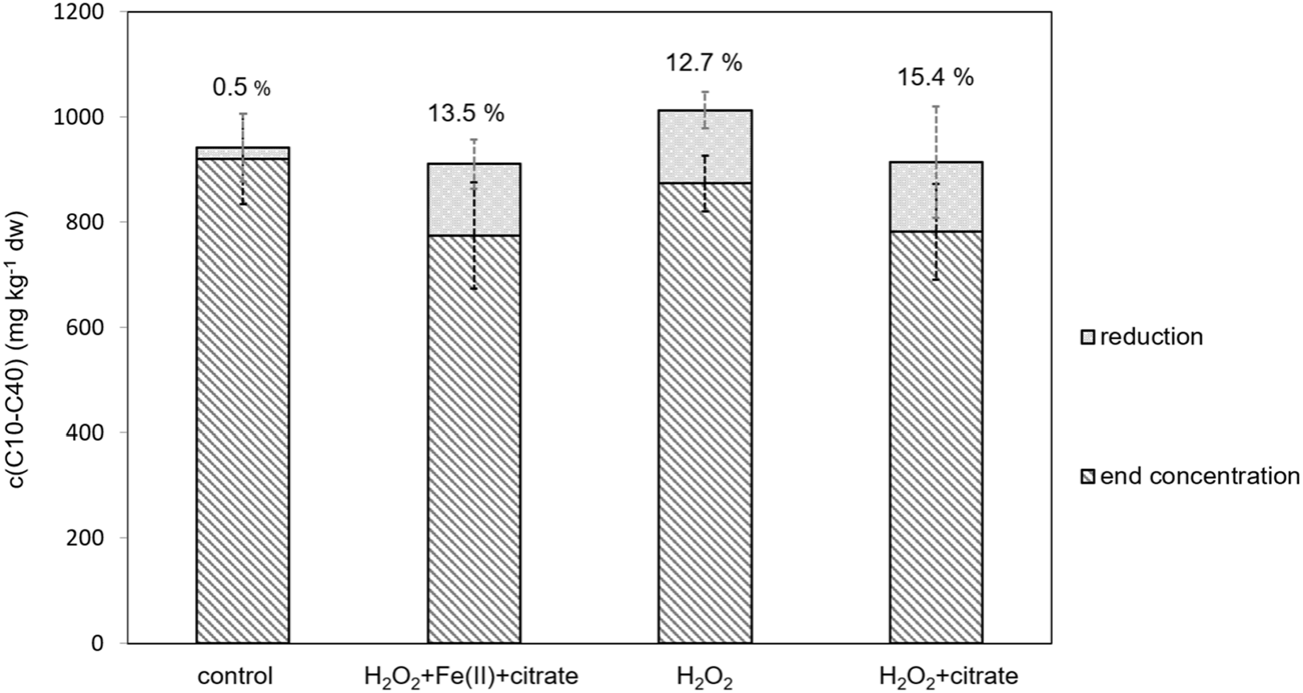
The effect of treatment on diesel oil hydrocarbon levels. The reductions (%) in oil hydrocarbon concentration (sample height weighed averages ± 85 % confidence interval) from samples before and after treatment from depth 0–100 cm in soil

Cleaner soil was used for the bottom layer, but since the exact oil hydrocarbon levels had not been tested prior to the treatment, this data was excluded from the comparison. These concentrations were in each case comparable between control and different chemical treatments (380 ± 30 mg kg^−1^ dw). In the three sampled layers between 0 and 100 cm from which data existed both from before and after treatments, approximately 13% reduction in oil hydrocarbon levels was observed in the treatment without citrate or chelate (Fig. 6). Based on the average values measured from the other two peroxide-treated lysimeters, even with visible differences in the reaction temperature, the reductions were of similar magnitude. As no significant physical transport of oil was observed either from the bottom soil or from the filtration water, peroxide treatment is suggested to have affected the oil hydrocarbon levels through chemical oxidation rather than mobilization alone. The initial mobilization effect on the soil hydrocarbons was, however, in all cases higher than the total reduction. Samples withdrawn from the bottom of the soil column did not have a detectable layer of insoluble oil hydrocarbons and oil hydrocarbon concentrations were detected only at low levels. This would suggest that mobilization was only a momentary effect and that the diesel was absorbed back into the soil once the reaction had waned.

## Discussion

In this study, the role of catalysts and citrate chelate in a hydrogen peroxide-based treatment of MTBE-spiked pore water was tested in different scales. When thorough mixing of the media could be achieved in small-scale trials, these additions were found to affect soil pH to such a degree that conditions were greatly altered in regard to catalyst solubility. In the laboratory scale tests with MTBE, Fe(II) as a catalyst was found to be preferable to Fe(III) since higher contaminant removal rate was achieved closer to neutral pH. The soil used in the lab test was a sand and gravel mix and therefore the benefits from higher acidity of Fe(III)sulfate would decrease with soils with higher buffering capacity, such as clayey silts. The differences in strength as acidifying agents relate to the differences in *Z*^2^*R*^−1^ ratio and as such the change in the oxidation state increases the strength of the ion as an acidifying agent. If working with lower grade products, the differences in strength can also relate to the purity levels of the crystals produced at different pH levels.

Also, with Fe(II), a smaller amount of catalyst would perhaps be required for a similar reduction rate. Matta et al. (2007, 2008) found that the Fe(II) content in soil was a key indicator of the performance level in both acidic and near neutral pH conditions. In some cases, Fe(III) has been stated to hold a higher catalytic potential than Fe(II) since hydrogen peroxide is being consumed in oxidizing Fe(II) to Fe(III) in non-radical producing reactions (Watts and Teel 2005). In the laboratory scale tests now performed, lag periods were found to result from lower additions of both peroxide and the catalyst, and differences between oxidation states were negligible by comparison.

As seen in the lysimeter scale, even if the conditions are otherwise made more favourable for chemical oxidation, use of additional catalysts may result in a trade-off. Readily available catalyst is likely to shorten the lag period before commencement of a vigorous reaction which will both consume the peroxide before the target depth and shorten the radius of impact. Injecting the peroxide and the catalyst independently would require more complex feeding systems to downplay some of these negative traits. Onsites with limited permeability where injections have to be performed over a longer period this would slow down the protocol and, in some cases, require a more concentrated dosage of the additives. Goi et al. (2009) also found that treatments with additional catalysts appear to be more toxic to soil biota than reactions catalysed by soil minerals alone.

The alkaline effect of chelates observed here was found to be one of the probable explanations for hampered reaction intensity (Pardo et al. 2014). If the solubility of Fe was positively affected by chelation, this effect was still outweighed by the negative effect of increasing alkalinity. In soil mineral, iron catalysed reactions; however, chelation had a moderate positive effect on the removal of MTBE from water within the first 24 h. Since, as seen in the lysimeter scale, additional catalysts complicate the injection procedure, the results favour chelation.

Citrate as a chelate has a documented ability to slow down peroxide consumption and to increase the efficiency of Fenton’s reaction-based chemical oxidation (Vicente et al. 2011; Pignatello et al. 2006). This may be beneficial since lessened reaction intensity may reflect a reduced consumption of H_2_O_2_ especially with regards to the non-radical producing reactions. In the lysimeter scale test with diesel-contaminated soil, citrate increased the pace of the reaction as observed from the temperature in the top soil and the reduction rate of peroxide. According to the results from the small-scale PID test with MTBE, this increasing intensity appears to contribute to the contaminant removal.

Experiments failing to achieve any significant stabilizing effect with the same amount of citrate have also been published (Innocenti et al. 2014). Moreover, even with an increased lifespan of H_2_O_2_, enhanced degradation of oil hydrocarbons cannot always be expected. Pardo et al. (2014) suggested that the contaminant and the citrate chelate could be in competing positions with regards to the added peroxide. The reactions between chelates and radicals may also prove counterproductive and the ligands formed can be absorbed to the soil (Vicente et al. 2011).

In the laboratory scale test, once the peroxide concentration was high enough (5 M), the differences in short-term removal efficiency between different treatments were to some extent balanced after 24+ hours. This may be because, with increasing doses, enough peroxide would be available for Fenton’s reaction to occur at sufficient efficiency even under suboptimal pH conditions. This is suggested to have been the case on some accounts (Goi et al. 2006). Another possible explanation is that increasing peroxide concentrations also enhance removal through an alternative mechanism, namely volatilization.

Elevated volatilization in relation to control studies was documented in scales 10 and 1000 L when soil acidity was not lowered below 5.5. In both cases, this was most apparent during the initial hours after the injection. In the 10-L test, a clear reduction in MTBE concentration in the gaseous headspace was achieved without the presence of chemical breakdown products. This presence would also be related to the degree of chemical mineralization achieved and the result cannot thus be considered to rule out chemical oxidation as a contributing mechanism altogether. These by-products have, however, been documented to appear in higher concentrations in neutral rather than acidic (pH 3) conditions, indicating a lower degree of mineralization (Khodadadi Darban et al. 2009). No similar peak in volatilization was observed in the 1-L scale, where reduction through some physical mechanism would have had to happen either after a lag period of 4 h or through continuous, moderately increased volatilization without a distinguishable peak.

These results suggest that volatilization is either cancelled out or covered under the primary mechanism of chemical oxidation, and that the effect would therefore be negligible in favourable conditions. According to Innocenti et al. (2014), when increased volatilization was observed at a MTBE-contaminated groundwater site within the same timeframe as the one described here, 99% of the total reductions could still be attributed to chemical mineralization. The H_2_O_2_ concentration then used (6%, 1.8 M) was close to the optimal for gas production as described by Baciocchi et al. (2010). In the site treatment (Innocenti et al. 2014) case, no additional catalysts were used but addition of peroxide was documented to have increased soil acidity from approximately pH 6 to pH 4, a level closer to the optimal pH range than the one described here.

For chemical oxidation, H_2_O_2_-contaminant mass ratios as high as 5–50:1 have been reported for soil and 1–5:1 for contaminant in water, by providers such as USP Technologies. These ratios are greatly affected by both pH and catalyst concentration (Goi et al. 2009). In our tests, the oxidant demand of the soil itself was found to be negligible in comparison with the mass of oxidants added with the relative strengths of hydrogen peroxide and hydroxyl radical as oxidizing agents taken into account. In the MTBE PID tests with the highest catalyst addition, a 26:1 H_2_O_2_–contaminant ratio was achieved (0.5 M H_2_O_2_, 88 % average reduction). With the 5 M dose of peroxide this ratio was 230–240:1 regardless of the treatment, which means that when the alternative effects are compensated through increase in peroxide dosage alone, the protocol is comparatively inefficient. However, in these cases, peroxide was added in excessive doses as in terms of peroxide-contaminant radios, as with mineral-catalysed reaction the lowest ratio was achieved already with 1 M dose of peroxide (72:1, 35% reduction).

In the pilot test, after two H_2_O_2_ injections, a ratio of 220:1 at highest had been achieved. The result indicate that the efficiency was similar regardless of the scale and also that within the tested range of concentrations, the total dosage could be injected in several smaller doses rather than the effect being achieved only when a certain threshold in the peroxide concentration had been reached. In the diesel lysimeter test, peroxide was added in approximately 40:1 mass relation to diesel and the measured reduction in the 13% range would result in 300:1 stoichiometry. This means that the reduction in efficiency for mid to heavy range hydrocarbons would to an extent relate to differences in the initial contaminant mass. Even without significant difference in these ratios, the choice of compound tends to affect remediation situations in these cases. With diesel-contaminated sites in need of remediation, the total mass of the compound is generally higher than with water-soluble ones. For example, the Finnish threshold values, the concentrations requiring risk assessment, for diesel and MTBE/TAME in soil are of a rather different magnitude, 300 and 0.1 mg kg^−1^ respectively (Reinikainen 2007). The relative importance of rebounds is hence associated with differences in the non-mineralized latent masses, as was seen in the lysimeter tests. As non-volatile compounds are more difficult to collect in situ, they are also more problematic. Within the restricted area of the lysimeter used here, transport of non-volatile fractions from soil to water did not result in horizontal mobilization as the aqueous phase would be absorbed into the soil column once all forming gases had escaped the pore space. Nevertheless, considering the duration of the reaction, this mechanism, when combined with horizontal movement of pore water could result in displacement of the contaminant within the treatable area. This could, according to the results, outweigh the actual remediation gain through chemical mineralization and increase the risk of contaminant mobilization.

As was found to be the case with MTBE-spiked water, in some situations with low total mass of the volatile contaminant, mineral catalysed treatment near neutral pH can still be considered. As described earlier, these are the conditions in which volatilization is also a factor. The production of gases is a temporal effect and removal of the contaminant from the unsaturated zone is affected by various factors. With compounds such as MTBE, with Henry’s constant higher than 2.65 × 10^−5^, the layer limiting the volatilization is between pore water and pore air in the soil column (Spencer et al. 1988; Caldwell et al. 2001). The volatilization from water to air is dependent on the balance of concentrations in both phases, and volatilization weakens with decreasing concentrations in the aqueous phase even if water is still being vapourized (Spencer et al. 1988). Once the reaction is over the balance between two matrixes will return resulting in a rebound effect (Krembs et al. 2010). We observed a similar phenomenon in the lysimeter scale with MTBE-spiked water, suggesting that in cases where volatilization adds to the total removal efficiency, peroxide-to-contaminant mass stoichiometry is no longer a relevant way to measure reaction efficiency as it will wane in the process. This phenomenon is, however, affected by the extent to which gases are able to escape the soil column and these types of rebound effects could therefore be minimized with sufficient soil vapour extraction techniques. In 10 L scale, SVE was not required for final MTBE concentrations of 8.8 mg L^−1^ in the aqueous phase, only slightly surpassing the 7.5 mg L^−1^ level considered unhazardous by the Finnish National Institute for Health and Welfare, whereas a similar level was not reached with the depth of the non-saturated zone in the lysimeter test. Also, as was found in the latter scale, with these margins, the effect of rebounds from the gaseous is to be considered as significant. The effect of SVE should therefore be further tested in situ, with sequential control periods, in an environment where appearance of NAPLs can be excluded with certainty, and where the role of chemical mineralization can be marginalized. Since lowering the soil pH to optimal is one of the primary technical difficulties in Fenton-based in situ chemical oxidation of soils (Goi et al. 2009), volatilization is suggested to play a role in most scenarios even when it is not targeted. To further study the observed effect in situ, high resolution monitoring of soil pH should be performed in connection with analyses on soil vapour formation and composition.

## Conclusions

Our study provided following insights to the questions outlined in the “Introduction”:

1. In each of our tests with MTBE-contaminated water hydrogen peroxide concentration was the main parameter associated with removal efficiency. Unfavourable changes in conditions for chemical mineralization, such as increase in soil pH, could be compensated for by increasing the hydrogen peroxide concentration. The benefits and hindrances associated with other additives were observed in connection to their acidifying or alkaline effects. Additional benefits not associated with changes in pH were Fe(II) being the preferable oxidation state for catalytic Fe near neutral pH and citrate having a positive effect on reaction intensity with soil mineral–catalysed reactions; however, neither of these effects was found to be statistically significant. Scaling up resulted in technical challenges in catalyst addition as hydrogen peroxide was being consumed before reaching the target depth.
2. When high removal efficiency was achieved with increase in hydrogen peroxide concentration, it was found to be connected to heightened importance of volatilization as a secondary mechanism. Since the effect of catalyst addition and pH were interconnected, this phenomenon cannot be traced to either parameter alone. However, what could be concluded was that volatilization played an increasing role in conditions non supportive to chemical oxidation, whereas with high degree of chemical mineralization this effect or its significance is reduced.
3. The secondary physical removal mechanism as observed can lead to increased total removal of a volatile contaminant but also to an increasing fluctuation between reduction and rebound from the gaseous phase, especially with lowering contamination levels. As seen in this study, the type of physical effect leading to heightened volatilization of MTBE is at least temporally mobilizing diesel from soil to the aqueous phase. It was also found that these reductions from temporal-physical transport at times outweighed the reductions through chemical mineralization. With both contaminants different type of trans phase transport was hence achieved resulting in fluctuation in the results from a single-phase monitoring. The effect of these trans phase rebounds on the overall success was different in each case, and the ways to reduce these rebounds would also differ. Even without soil vapour extraction, injection of high concentrations of hydrogen peroxide was found to be an applicable method to reduce the level of MTBE contamination, with the chosen soil type and initial MTBE concentration in conditions unfavourable for chemical mineralization. Whilst only temporal transport of diesel size fractions into the aqueous phase was observed here, in situ this mechanism could be suggested to increase the risk of groundwater contamination associated with the treatment. This can reduce the potential of the technique to work as a multi-contaminant treatment method, and also to add to the risk level in cases where only volatile compounds are targeted as the primary risk factor in a multi-contaminant environment.
